# Checking Perspiration

**Published:** 1873-07

**Authors:** 


### CHECKING PERSPIRATION.

A MAN walked rapidly along the street on a warm day, and while perspiring stepped into a barber's shop to be shaved, and pulling off his coat waited for his turn; but the room was cool, a chill sensation came over him before the barber completed his operations; a fever followed, then inflammation of the lungs, ending, in a few days, in the death of the good and universally loved Bishop McIlwaine.

About the same time, and not far away, another man became overheated after a longer walk than usual, and being a little tired and over warm "he sat down a few moments to enjoy the fresh afternoon breeze," by which perspiration was checked too soon, bringing on a rheumatic catarrh, affecting also the back and hips, and producing sciatic irritation in one leg. The use of leeches was advised in the first instance, but fortunately the suggestion was not acted on, in consideration of the Pope's lymphatic constitution, which, at his advanced age, could hardly rally from any considerable loss of blood. His Holiness was more prudently rubbed with anodynes and poulticed with mustard, which method of cure, by soothing the local pains, reduced the fever, which was never high, and enabled the venerable patient to repose tolerably easily in bed, although, during the first few days, any attempt to get up or to move about brought on the pains again. Thus it is that the lives of the most eminent are imperiled and even lost by inattention to little things, which it would seem any one of ordinary intelligence would guard against. And yet it is in this way that multitudes every year lose health and life, and ten to one the reader will lose his own life in the same way, by cooling off too soon after exercise. The "Roman Fever," so deadly, is often occasioned by persons getting a little warm in walking in the streets and then going into a picture gallery, many degrees colder, and, being interested in the paintings, time passes and they become chilled before they know it. Always throw on an extra covering on entering these places.

